# The Relationship Between Ethical Leadership and Employee Job Satisfaction: The Mediating Role of Media Richness and Perceived Organizational Transparency

**DOI:** 10.3389/fpsyg.2022.885515

**Published:** 2022-05-18

**Authors:** Kaimeng Guo

**Affiliations:** Department of Communication, Faculty of Social Sciences, University of Macau, Taipa, Macau SAR, China

**Keywords:** ethical leadership, media richness, perceived organizational transparency, organization sustainability, psychology, employee satisfaction

## Abstract

Ethical leadership (EL) is a vital component driving firms’ practice, significantly affecting employee satisfaction (ES). The objective of this study was to investigate the relationship between EL and employee job satisfaction. Moreover, the study investigates the mediating effect of media richness (MR), perceived organizational transparency (OT) on EL, and ES. In addition, the study used a convenient sampling technique for collecting the data from 276 employees working in the service sector of Macau. Essentially, questionnaires had used as the fundamental tool guiding the data collection method. The findings indicate a positive and significant relationship between EL, ES, MR, and perceived OT. The MR and perceived OT have significant mediating effects between EL and ES. The study is beneficial for the service sector of Macau to plan the strategies for their employees in terms of organizational sustainability and success. The study is also helpful for the government to understand EL and for the researcher to search the new aspects of EL in organizations for future research direction.

## Introduction

In today’s business world, moral ethics have gained considerable popularity in the management literature. At present, emerging enterprises have significantly extended their role from achieving organizational prosperity to bringing colossal individual benefits. The developing notion has brought immense advantages for worldwide businesses, compelling them to adopt moral and ethical considerations as an essential tool for organization’s welfare. Perhaps, to fulfill the progressing demand of workplace ethics, the study demands both the public and private organizations to embrace the concept of ethical leadership (EL) in achieving the global socio-economic boom (Deal, 2018).

Comprehensively, EL refers to adopting moral conduct, causing an individual to initiate the two-way relationship, communication, and decision-making (Brown et al., 2005). Ethical leadership enhances organizational performance by integrating moral values into organizations’ practices. Ethics plays a critical role in developing the right set of ideas, thoughts, and principles, influencing individuals’ work behavior, conduct, and actions (Rabie and Abdul Malek, 2020). In particular, the resilience of leaders’ character requires consistency with moral virtues. Good leadership demands not only managers’ expertise, knowledge, and strategic power but also leaders’ moral character. Given the statement, the moral characteristics of EL reveal leaders to be morally responsible, visionary, and honest (Sharma et al., 2019). Nonetheless, this moral aspect empowers the employees’ behavior, thus influencing their motivation, performance, and satisfaction.

Moral leadership is a unique concept assisting the employees’ work, subsequently raising employee satisfaction (ES). Employee satisfaction is a pleasurable emotion (i.e., psychological and social) related to employees’ achievement (Teimouri et al., 2018). Ethical leadership vigorously accelerates employees’ productivity, thereby reporting greater satisfaction (Freire and Bettencourt, 2020). The comprehensive effect of EL extends far beyond influencing employee behavior to satisfy the individual’s needs and desires. The study shows that EL guides employees’ behavior, actions, and attitudes, thereby increasing their work contentment (Kaffashpoor and Sadeghian, 2020).

Moreover, firms’ communication also plays an integral role in increasing the followers’ satisfaction. In this perspective, media richness (MR) is a crucial factor driving organizational communication. MR alludes to the leaders’ ability to integrate innovation (e.g., information technology) for delivering personalized information, feedback, and messages (i.e., verbal and non-verbal cues). In particular, the study shows that MR strengthens an organization’s communication network, thereby fostering workplace interactions, cognition, and satisfaction (Sheer, 2020). The study suggests that leaders choose the most effective communication media channel for supporting organizational transparency (OT) (Sommerfeldt et al., 2019).

Information discrepancy influences the organizations’ performance, thus affecting the stakeholders’ attitude (e.g., actions, motivation, and satisfaction) (Losada-Otálora and Alkire, 2019). Consequently, maintaining OT in business practices (e.g., information sharing and communication) is vital for successful business functioning. OT refers to the firm’s intention of sharing the business information (i.e., positive and negative) with its stakeholders (e.g., internal and external). It includes information about organization’s practices, attributes, procedures, and actions. The study states that the organization is responsible for sharing truthful, honest, correct, and accurate knowledge (Sarfraz et al., 2019; Shah et al., 2019; Schnackenberg et al., 2021). Hence, the management should promote higher disclosure of information, awareness, and ownership for achieving greater business transparency.

Unfortunately, the deficiency in literature had conceptualized a backdrop in understanding EL as a fundamental phenomenon influencing the employees’ satisfaction. This underlying mechanism stretches beyond the boundary of traditional management to the moral configuration of leadership, potentially increasing ES. Hence, comprehensive study reviews on EL require consolidation as the prior studies lack a workable structure guiding the direct relationship between the terms. Accordingly, the research shows that the relationship between EL and ES is limited (Moon and Jung, 2018; Ajaz et al., 2020). Furthermore, despite the increasing role of novel technology, the research states that MR (Ishii et al., 2019) and OT (i.e., leadership knowledge sharing) had also remained silent under the moral lens of EL (Bavik et al., 2018).

Significantly, to fill the research gap, the study empirically conceptualized the effect of EL on ES (Vlachos et al., 2013), fundamentally in the light of moral leadership theories. It also investigates the direct impact of EL on MR and OT. Moreover, the study framework explains the connection between MR and OT with employee job satisfaction. Finally, it highlights the mediating role of MR and OT, bridging the nexus between EL and ES. In particular, this study theoretically addresses all the research gaps by presenting a solid knowledge foundation on the effect of EL on ES, MR, and OT.

Potentially, this study is a unique contribution to bridging the prior research gaps. Therefore, based on this statement, EL in the hospitality sector has gained most researchers’ attention, thus elevating its need for investigation (Kabene et al., 2006). Leaders play a prime role in enhancing the health service, subsequently facing immense pressure to behave ethically, influencing the employees’ performance satisfaction (Shafique et al., 2018). Accordingly, this study holds paramount importance by allowing future researchers to investigate the fundamental relationship between EL and employee job satisfaction. Furthermore, the prior literature states that EL had explored various times in context to several mediating variables [e.g., moral identity (Arain et al., 2017), psychological contract (Ahmad et al., 2019), and behavior outcomes (Khan N. et al., 2018)]. Hence, this unique study highlights two significant variables leading to improved job satisfaction.

Perhaps, this study has a broad scope in the management and psychological literature. It develops a solid foundation, highlighting the increasing demand for moral practices. The underlying mechanism of EL has accumulated the fragmented literature from management discipline, thus providing a clear picture of morality in leadership. Indeed, to the best of our knowledge, this novel study integrates the dispersed literature on EL under one roof. Perhaps, to cover the literature deficiency, the study provides the management experts with essential knowledge about the dynamic nature of moral leadership and its increasing effect on ES.

Essentially, this study had divided into various sections. This article begins by briefly introducing the study variables in the light of relevant theoretical studies. Then, the “Literature review” section (i.e., literature review) gains the reader’s attention by proposing a set of testable hypotheses, formulating the direct and indirect relationship between the variables. Moreover, the “Methodology” section suggests the most relevant tools used for data analysis, and the “Results” section significantly illustrates the study outcomes. Finally, the “Discussion” section discusses the study outputs, and the “Conclusion” section concludes the research article.

## Literature Review

Significantly, the evolution of modern leadership and advancement in communication technologies have shaped the organizations’ working culture, processes, and structures, thereby bringing numerous advantages for the leaders and the employees. Accordingly, the “Literature review” section aims to investigate the fundamental relationships in the light of the moral leadership perspective. Along with this, it also presents the role of the leaders in developing a favorable working climate, work practices, and behavior, thus enhancing the employees’ satisfaction. Indeed, this section elaborates the relevant literature review in the following terms: EL, ES, MR, and OT. All the variables and terminologies had demonstrated in the same series in the section below.

### Ethical Leadership and Employee Satisfaction

Over the years, EL has received massive popularity from scholars, researchers, and managers, essentially ensuring the application of the various definitions of moral leadership in the work setting (Ko et al., 2018). EL refers to the behavioral approach of promoting normative conduct and actions (Brown et al., 2005). Its definition establishes a distinct association among the workers, potentially improving their work satisfaction. Given the illustration, the research indicates that advanced leadership enhances organizational outcomes by reporting increased employees’ commitment and job satisfaction (Qing et al., 2020; Abdullah et al., 2021).

In leadership, moral ethics is a vital component driving firms’ practices. Leadership ethics develop a critical guide for the organizations’ members, thus encouraging them to follow moral cues. Ethical leaders support the integration of moral principles, thus making leadership integrity, fairness, and empowerment improve ES. The positive consequences of leadership ethics strengthen the employees’ attributes, thereby influencing employee cognition and satisfaction. In line with the statement, the research indicates that leaders’ moral characteristics demonstrate high consideration for employee well-being, subsequently nurturing employee workplace satisfaction (Moon and Jung, 2018).

In addition, moral leaders hold a powerful position in stimulating organizational behaviors. The leadership characteristics reinforce a positive attitude in employees, guiding their work actions. EL effectively influences employee actions by tapping into the employees’ values. The employee-leader congruence makes the employee practice moral values, thereby promoting ES in the workplace. Given the explanation, the research reveals that leaders exhibiting high ethical standards direct the employees’ work behaviors, subsequently raising their job satisfaction (Abdullah et al., 2018; Freire and Bettencourt, 2020). Indeed, ethical leaders play an instrumental role in enhancing the employees’ work behavior. Ethical leaders motivate individuals to work harder toward the organization’s development (Charoensap et al., 2019; Sarfraz et al., 2022). Given the articulation, the study shows that the leaders enhance employees’ satisfaction, thus making the employees conducive to working hard in the challenging environment (Cleveland et al., 2019). In particular, leaders’ ethical conduct is an effective predictor of ES. The literature concludes that EL positively influences the employees’ behavior, satisfaction, and performance (Shafique et al., 2018). Consequently, based on these research findings, we have suggested the following hypothesis:

*H1: EL has a positive and significant impact on ES.*

### Ethical Leadership and Media Richness

In the 21st century, innovative media tools have become crucial for sharing moral information with employees. With the increasing leadership ethics, the prior literature indicates that the accelerating media channels foster moral teachings, thus shaping the leadership attributes (Roman et al., 2019; Mohsin et al., 2022). Recently, the growing EL has alleviated the need for innovative technological tools guiding business practices. At present, the developing significance of media channels has enabled businesses to incorporate novel digital technologies for establishing an effective communication network. This complementary mode of connection significantly benefits today’s employees, leading the media-based channels to influence work structure. Essentially, an EL style brings immediate results to the organization’s performance. The moral leadership style empowers the workers to benefit from the increasing advantages of MR, thereby supporting the organization’s communication process (Bhatti et al., 2020).

The unique communication technologies make the MR play a critical role in improving an organization’s understanding of information. Ethical leaders use effective media channels to share moral knowledge with their employees. High MR enables the leaders to send personalized messages to the employees regarding organizational matters and activities. Organizations utilize multiple communication channels for connecting with its stakeholder (i.e., business partners, employees, and customers). In leader-subordinate communication, the media-based tools foster the organization’s communication process. The most effective media-enriched communication channels include email, Facebook, Twitter, and Google Docs (Aritz et al., 2018). Given the articulation, the research shows that the more efficient the media channel is, the more ethical leaders influence team cohesion through moral cues (Sedrine et al., 2021).

Indeed, recent technological progress has led the organization to experience a new paradigm of working in real space, thereby radically affecting people’s behavior, actions, and communication. EL influences organizational communication, thereby developing a moral-ethical climate. The moral character of ethical leaders empowers the employees by providing them with an environment where they can practice moral teaching. Given the illustration, the study states that ethical leaders adopt efficient media tools for educating employees about business ethics (Wu, 2021). However, MR depends on the choice of the leader who prefers to cascading down the ethical information to the low-level workers. The research suggests that social media applications are the most popular sites for generating, consuming, and dismembering information across the business network (Bashir et al., 2021). Consequently, based on the previous literature, the hypothesis indicates EL to strengthen the MR, thereby proposing the following hypothesis:

*H2: EL has a positive and significant impact on MR.*

### Ethical Leadership and Organizational Transparency

Due to the rapid globalization, organizations today are functioning in an environment where consumers’ demands are constantly changing, escalating its intensity on the organization’s practices. An organization operating in a higher competitive environment alleviates the need for adopting transparent business initiatives in response to market changes. Undoubtedly, in recent times, OT has become a critical instrument for leaders in adapting to changing environments. Ethical leadership shapes the organization’s culture, leading the business transparency to reduce business uncertainty (Metwally et al., 2019).

According to the definition, OT refers to the firm’s intention to transmit business information (i.e., negative and positive) to its stakeholder (Schnackenberg et al., 2021). Significantly, to maintain information transparency, moral leaders should look after the interest of both the internal and external stakeholders. Integrity, one of the crucial leadership traits, effectively motivates the leaders to share truthful information with firms’ stakeholders, subsequently enhancing organizational value and employee behavior (Ete et al., 2021). In particular, this accountability dimension of data transparency assists the employees’ actions by communicating the change information to the intended user (Yue et al., 2019). Consistently, leaders’ characteristics had been constructed around the virtue of fairness, accountability, honesty, and integrity (Francis et al., 2018). Perhaps, business ethics reflected in the leadership makes the leaders’ integrity develop an open communication system, guiding the individual moral behavior. In particular, knowledge sharing is a significant aspect of guiding the employees’ behavior. Given the illustration, the study shows that ethical leaders provide moral information to employees, thus making OT enhance employees’ performance (Bhatti et al., 2020).

Furthermore, from the organization’s perspective, knowledge flow is vital for business success. Perhaps, to improve individual’s performance, the leaders should provide ethical teaching to the employees by adopting different media channels. In recent years, various platforms have enhanced the flow of moral knowledge to the employees (e.g., social media and Facebook). Perhaps, the study suggests leaders utilize the competence of emerging MR for staying connected to the employees (Braojos et al., 2019).

Arguably, EL cannot function without an effective communication structure. A transparent communication network encourages the leaders to develop a two-way communication process, thus enhancing the firm’s communication (Jiang and Luo, 2018). Ethical leaders play an active role in directing the transparent communication environment. Given the explanation, the study shows that unethical climate decreases the influence of moral leadership, thereby devastatingly affecting the organization’s outcome and employees’ behavior (Halbusi et al., 2021). Undoubtedly, leaders play a significant role in building the organization’s ethical climate with maintaining OT. Consequently, the literature suggests the following hypothesis:

*H3: EL has a positive and significant impact on OT.*

### Media Richness and Employee Satisfaction

As the organizational environment continues evolving, new technologies have rapidly emerged as an efficient tool for reaching the employee. Advanced media technologies have ensured the development of managerial communication between leaders and employees. Social media channels significantly facilitate the organization’s communication process, thereby enhancing the individual’s job performance. In support, the study refers to videoconferencing as an innovative tool driving the firm’s communication (Lee et al., 2018) and employees’ satisfaction. Given the statement, the new influx of MR influences the organization’s communication with fostering ES (Chen and Chang, 2018).

In addition, concerning management literature, ES alludes to the pleasant feeling of happiness. The firm’s communication ensures the potential use of media channels for acquiring favorable individual outcomes. In particular, deciding on the most appropriate media channel, the managers should take care of the employee preference. Media choices influence the employees’ satisfaction. Complex media platforms are hard to operate by employees. In the illustration, the study shows that media channels, such as email and telephonic calls, are fundamental tools driving employees’ satisfaction (Erjavec et al., 2018). Perhaps, due to the increasing significance of media quality, the management needs to consider the interest of the employees in selecting the best media tool (Verčič, 2019), subsequently enhancing employees’ satisfaction.

Fundamentally, scholars and professionals have potentially recognized the significant role of MR in enhancing employees’ job satisfaction. MR plays a crucial role in setting the tone of the firm’s communication process. Consistently, the prior literature shows that MR enhances the employees’ satisfaction. In explaining this notion, the study states that MR (e.g., online platforms) increases individuals’ job satisfaction, thereby improving task accomplishment (Fleischmann et al., 2020). Certainly, MR nurtures the employees’ morale and satisfaction. It ensures the exchange of verbal and non-verbal signals, messages, and texts with the end user (Mehra and Nickerson, 2019). It provides a framework for transmitting data with the stakeholders (i.e., internal and external). The increased MR helps the individual acquire the essential data for accomplishing the work task. Hence, the study shows that the improved relatedness of media channels positively increases ES (Khan I. U. et al., 2018). Therefore, the literature concludes the following hypothesis:

H4: *MR has a positive and significant impact on ES.*

#### The Mediating Role of Media Richness

In the increasing era of globalization, the technological advancement in information technology has fostered EL to bring colossal benefits to individuals. At present, organizations have embraced novel technologies for performing business functions. The progressing globalization has massively compelled the organizational leaders to connect with the employees through advanced technological networks. However, this developing technological reliance had demanded ethical leaders to foster ethical behavior in speeding up the business performance.

Leaders’ communication behavior is an essential component in supporting individual outcomes. Ethical leaders use multiple communication methods to convey the organization’s vision, messages, and teachings, thus establishing a positive leader-subordinate relationship. Ethical leadership encourages adopting modern technologies, subsequently enhancing organizational communication and ES. Accordingly, the study states that firms’ embracing technological innovation builds an immediate leader-employee connection, thereby improving employee trust and satisfaction (Tkalac Verčič and Špoljarić, 2020). The prior study shows that social media has become a vital communication tool for sharing data (Khan et al., 2019). Social media usage enhances the employees’ ability to respond to workplace challenges. It establishes a leader-follower connection, thereby fostering employees’ satisfaction (Bhatti et al., 2020).

In the EL paradigm, MR strengthens individual work outcomes by improving job satisfaction (Chen et al., 2021). Employee satisfaction is highly dependent on employees’ choice of media quality. Employees prefer to use the potent media channel for communication with leaders (Aritz et al., 2018). In EL, a leader’s goal is to fulfill employees’ needs (i.e., information). Accordingly, moral leadership supported by computer-mediated technologies satisfies the employee’s desires for interpersonal communication (Braun et al., 2019). Therefore, by focusing on the communication richness, the study illustrates that today’s media channel affects the communication process of organizations, thus significantly influencing their employees’ satisfaction (Özden et al., 2019). Indeed, the study shows that ethical leaders promote adopting novel technologies for building a positive communication network that increases ES (Mehra and Nickerson, 2019). Perhaps, based on the previous studies, the literature suggests that MR should be a significant factor mediating the relationship between EL and ES.

*H4a: MR significantly mediates the relationship between EL and ES.*

### Organizational Transparency and Employee Satisfaction

In today’s digital world, OT has become the top priority of management leaders. It alludes to data sharing with creating an open organizational structure to promote business culture. Transparency is a fundamental attribute of the organization’s culture that guides the business practices, behaviors, activities, and practices. OT alone cannot meet the interest of the stakeholders. Therefore, management must understand the role of maintaining transparency in catering to the employee demand for information (Mappamiring et al., 2020) for directing the business practices, procedures, and activities. Firms’ accountability refers to their obligation of sharing the data with employees, thus guiding their behavior and satisfaction. Employee participation remains high when the organization presents workers with transparent data. The study shows that OT strengthens the employees’ engagement, thus fostering employee performance and satisfaction (Hofmann and Strobel, 2020).

Indeed, OT is a powerful driver of ES. ES largely depends on the information available to them. Information transparency encourages the firms to share transparent, truthful, and accurate information, thus assisting the employees’ activities. Concerning the firms’ communication, exposure to false and inadequate data creates confusion for employees, thus making it difficult for them to manage the flooding of unnecessary and overburdened data (Yue et al., 2019). To perform the work task, the firm must provide relevant knowledge to employees concerning the business content, purpose, and process. In particular, without the information transparency, it becomes hard for the employees to meet the performance standards, subsequently decreasing workers’ morale and workplace satisfaction. Hence, the organizations need to take care of data transparency for gaining optimum individual results (i.e., work satisfaction) (Ridwan et al., 2018). Moreover, the study shows that the organizations’ communication guides the individual’s work outcomes (e.g., reduced stress and problems), thus making this an entry point for the leaders to boost employee enthusiasm and job satisfaction (Sariwulan et al., 2019). Hence, based on the reviews of previous scholars, we have developed the following hypothesis:

*H5: OT has a positive and significant impact on ES.*

#### The Mediating Role of Organizational Transparency

Along with the transition from traditional management to EL, the leadership mechanism had constructed around the significant concept of OT, thus improving the business culture. EL plays a critical role in formulating business transparency in the communication network. Business information assists the employees in altering their behaviors, conducts, and actions. The organization’s morality holds a high potential for promoting openness and transparency of information. Accordingly, EL ensures the transfer of moral data to the workers, thus fulfilling their desire for progress. Given the statement, the study shows that EL is the direct way to increase OT and ES (Moslehpour et al., 2018).

The leadership characteristics emphasize adopting ethical principles, thus developing an environment for accelerating employee career satisfaction (Moon and Jung, 2018). Visionary leaders create an ethical environment, thus increasing the employees’ excitement and work satisfaction (Özturk and Senanur, 2021). Organizational transparency is critical to developing an ethical climate in the organization. EL ensures the establishment of moral and transparent business practices, procedures, and communications, subsequently achieving individual outcomes. The ethical environment enhances the employees’ perspective, thereby recording a positive response between EL and ES (Halbusi et al., 2021). Indeed, for an organization’s success, maintaining business transparency has become vital for leaders. The common goal of the organizations is to accelerate the employees’ satisfaction and morale. Therefore, to achieve this goal, leaders’ should create value for their employees by building a transparent organizational climate, thus influencing their work engagement and satisfaction (Meynhardt et al., 2020). Accordingly, the research states that the influence of ethical leaders on ES ensures the creation of the organization’s ethical culture, substantially internalizing the moral beliefs to satisfy the employees’ needs (Taştan and Davoudi, 2019). Therefore, the prior literature presents the following hypothesis:

*H5a: OT mediates the relationship between EL and ES.*

## Methodology

The quantitative study examined the direct relationship between EL, MR, OT, and ES. Moreover, the study also examines the mediating relationship between MR and OT and between EL and ES. The data are collected by the service sector of Macau. First-line managers, middle-level managers, senior-level managers, and executives working in the service industry participated in this study. The study adopted ten-item measurement scale of EL from the study by Elçi et al. (2012), and the four-item MR measurement scale was adopted from the study of Ben Sedrine et al. (2020). The four-item measurement scale of OT was adopted from the study of Rawlins (2008). The four-item ES measurement scale was adopted from the study by Chi and Gursoy (2009). The convenient sampling was used in this study, and data were collected by employees electronically. A total of 350 questionnaires are distributed to respondents and 290 questionnaires are received back; after checking the data, 267 questionnaires are finalized with a 76% response rate to analyze the data. The Statistical Package for the Social Sciences (SPSS) software was used for the data analysis.

## Results

Table 1 provides the complete details of the demographic characteristics of respondents who participate in this study. In the gender section, 161 respondents are men with 60.3%, while 106 respondents are women with 39.7%. Regarding age, 20 respondents age between 19 and 30 years with 7.5%, 78 respondents age between 31 and 40 years with 29.2%, 63 respondents age between 41 and 50 years with 23.6%, 77 respondents age between 51 and 60 years with 28.8%, and 29 respondents age more than 60 years with 10.9%. In the education section, 38 individuals are intermediated with 14.2%, 93 individuals have bachelor’s degree with 34.8%, 100 individuals have master’s degree with 37.5%, and 36 individuals have MPhil/other degrees with 13.5%. Moreover, in marital status, 46 individuals are single with 17.2%. At the same time, 221 individuals are married with 82.8%; in designation, nine individuals are accountants with 3.4%, 11 individuals are first-line managers with 4.1%, 107 individuals are middle-level managers with 40.1%, 111 individuals are senior-level managers with 41.6%, and 29 individuals are executives with 10.9%.

**TABLE 1:** Demographic characteristics.

Items	Frequency (*N* = 267)	(%)
**Gender**		
Male	161	60.3
Female	106	39.7
**Age**		
19–30	20	7.5
31–40	78	29.2
41–50	63	23.6
51–60	77	28.8
> 60	29	10.9
**Education**		
Intermediate	38	14.2
Bachelor	93	34.8
Master	100	37.5
MPhil/Others	36	13.5
**Marital Status**		
Single	46	17.2
Married	221	82.8
**Position**		
Accountant	9	3.4
First line manager	11	4.1
Middle level Manager	107	40.1
Senior level manager	111	41.6
Executive level	29	10.9

### Common Method Bias

**FIGURE 1:**
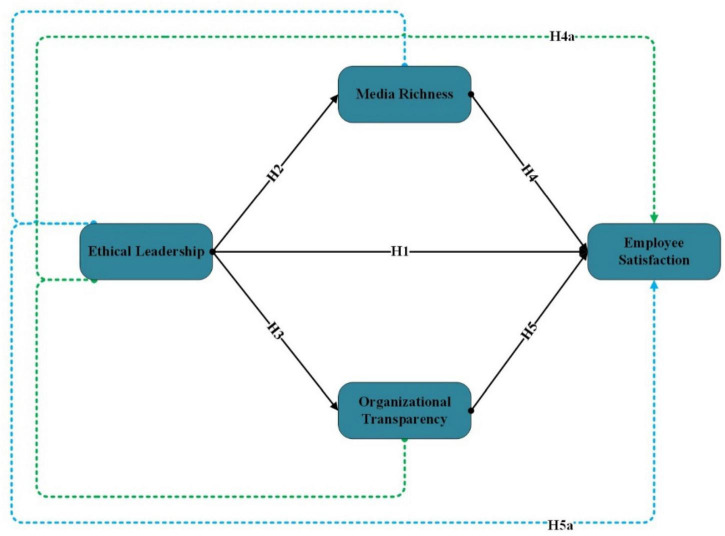
Conceptual framework.

**FIGURE 2:**
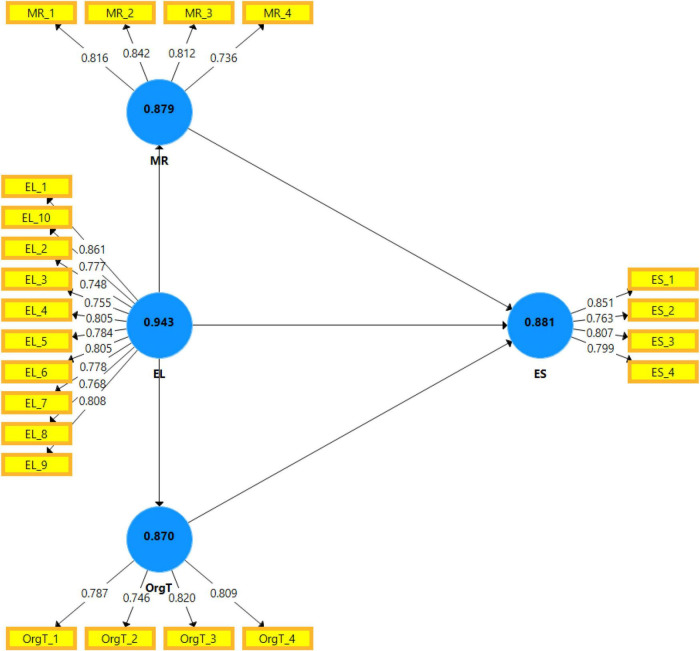
Graphical representation of assessment of measurement model.

This research also applied the common method bias using Harman’s single-factor approach (Figure 1). The variance extracted by one single factor is 10.235%, which is less than 50%, indicating no common method bias in this study (Podsakoff et al., 2003). Table 2 provides the detail of the assessment measurement model that shows the reliability and validity analysis. Cronbach’s alpha, loadings, and composite reliability should be greater than 0.70 (Tabassum et al., 2020), and the study meets the reliability threshold criteria. Therefore, the average variance extracted (AVE) should be higher than 0.5 (Sarstedt et al., 2019); the study meets the threshold criteria of 0.5. The reliability has been shown in this study. Figure 2 shows the graphic demonstration of the assessment measurement model.

**TABLE 2:** Reliability and validity analysis.

Construct	Items	Loading	A	CR	AVE
Ethical leadership	EL_1	0.861	0.943	0.943	0.623
	EL_2	0.748			
	EL_3	0.755			
	EL_4	0.805			
	EL_5	0.784			
	EL_6	0.805			
	EL_7	0.778			
	EL_8	0.768			
	EL_9	0.808			
	EL_10	0.777			
Media richness	MR_1	0.816	0.879	0.878	0.644
	MR_2	0.842			
	MR_3	0.812			
	MR_4	0.736			
Organizational employee	OrgT_1	0.787	0.870	0.870	0.626
	OrgT_2	0.746			
	OrgT_3	0.820			
	OrgT_4	0.809			
Employee satisfaction	ES_1	0.851	0.881	0.881	0.649
	ES_2	0.763			
	ES_3	0.807			
	ES_4	0.799			

### Assessment of Measurement Model

Table 3 shows the discriminant validity between constructs; there are two methods to measure the discriminant validity, namely, Fornell–Larcker and HTMT. The Fornell–Larcker method shows that the values on the diagonal represent the square root of the AVE, while the off diagonals are correlations. HTMT is the most reliable method to analyze the discriminant validity that should be less than 0.85 (Hair et al., 2016, 2019).

**TABLE 3:** Discriminant validity analysis (Fornell–Larcker and HTMT).

Constructs	1	2	3	4
Ethical leadership	0.789	0.685	0.553	0.557
Media richness	0.687	0.806	0.579	0.539
Organizational employee	0.554	0.58	0.803	0.341
Employees satisfaction	0.557	0.54	0.343	0.791

*Values on the diagonal represent the square root of the average variance extracted, while the off diagonals are correlations.*

The study meets the threshold criteria of HTMT and Fornell–Larcker; there is no discriminant validity issue between constructs. Table 4 demonstrates the variance influence factor, which shows multicollinearity by constructing its means and independent variables that are influenced by other variables in this study. The variance influence factor should be less than 10, which shows the acceptable values in this study, but in some cases, it can be less than 5 or 2.5, and the minimum range is 1. This study meets the threshold criteria; all variables have multicollinearity. Figure 2 shows a graphical representation of the assessment of the measurement model.

**TABLE 4:** Variance influence factor.

Constructs	1	2	3	4
1. Ethical leadership		1.852	1	1
2. Media richness				
3. Organizational employee		1.447		
4. Empolyee satisfaction		1.454		

### Structural Model

#### Hypothesis Testing

Table 5 indicates the direct relationship between variables. According to the beta valve, variables considered positive and negative relationship between each other, while a *t*-value threshold higher than 1.96 and *p*-value less than 0.01 mean that there is a significant relationship between constructs. In this study, hypothesis 1 indicates that the beta value is 0.413 and *t*-value is 4.209, showing the positive and significant association between constructs EL→ES. Hypothesis 2 shows that the beta value is 0.554, and the *t*-value is higher than 1.96; there is a positive and significant relationship between constructs EL→MR. Therefore, hypothesis 3 indicates that the beta value is 0.557 and the *t*-value is 8.295, higher than 1.96, showing the positive and significant association between constructs EL→OrgT.

**TABLE 5:** Hypothesis testing direct effect.

Hypothesis	Direct	Std.	Std.	T	P
					
	Relationships	*Beta*	Error	Values	Values
H1	EL→ES	0.413	0.098	4.209	***
H2	EL→MR	0.554	0.067	8.301	***
H3	EL→OrgT	0.557	0.067	8.295	***
H4	MR→ES	0.277	0.082	3.380	**
H5	OrgT→ES	0.215	0.069	3.089	**

*Indicates significant paths: p < 0.01, NS = not significant.

Moreover, hypothesis 4 finds that the beta value is 0.277, and the *t*-value is 3.380. There is a positive and significant connection between constructs MR→ES. Hypothesis 5 shows that the beta value is 0.215, and the *t*-value is 3.089, confirming the significant relationship between constructs OrgT→ES.

**FIGURE 3:**
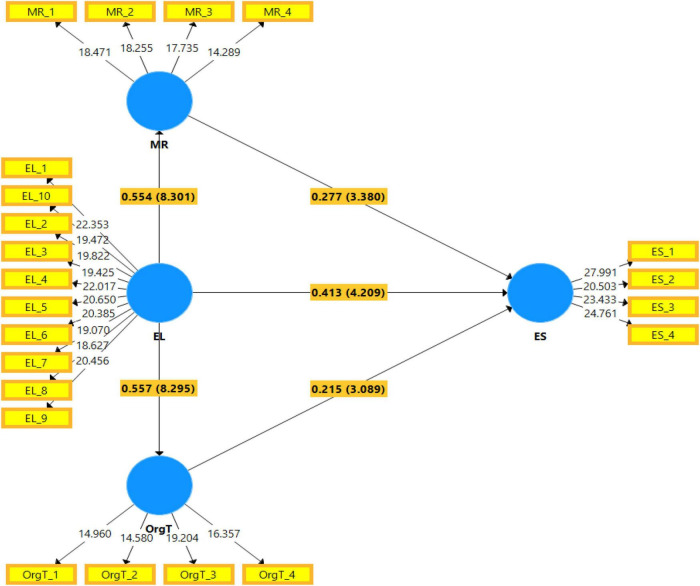
Graphical representation of the structural model.

Table 6 indicates the mediating relationship between MR and OT and between EL and ES. The results show that hypothesis 4a shows that the beta value is 0.154 and the *t*-value is 3.09, confirming the significant mediating relationship between EL→MR→ES. Hypotheses 5a confirmed that the beta value is 0.12 and the *t*-value is 0.044; there is a significant mediating relationship between EL→OrgT→ES. Figure 3 demonstrates the pictorial representation of the structural equation model.

**TABLE 6:** Hypothesis testing mediation effect.

Hypothesis	Indirect	Std.	Std.	T	P
					
	Relationships	*Beta*	Error	Values	Values
H4a	EL→MR→ES	0.154	0.05	3.09	**
H5a	EL→OrgT→ES	0.12	0.044	2.712	**

*Indicates significant paths: p < 0.01, NS = not significant.

### Quality Criteria

**FIGURE 4:**
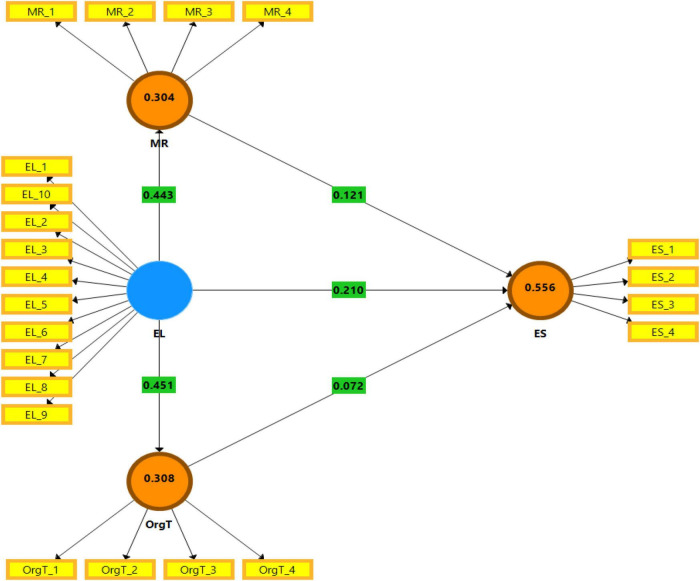
Graphical representation of R^2^ and F^2^ values.

Table 7 shows that the quality of the model, namely, adjusted R^2^, Q^2^, and F^2^ measured the quality. Therefore, the adjusted R^2^ shows the variability and close relationship of constructs in the model. The relationship between variables depends on the number of variables, either greater or lower. The higher values show a strong association between variables. The study has R^2^ values of ES, MR, and OrgT, which are 0.561, 0.307, and 0.311, respectively. Furthermore, the adjusted R^2^ values of ES, MR, and OrgT are 0.556, 0.304, and 0.308, respectively, confirming the strong relationship between the study variables. Q^2^ reveals that the predictive relevance of the model should be greater than 0; Q^2^ values of ES, MR, and OrgT are 0.342, 0.185, and 0.181, respectively, confirming that the model has good predictive relevance. The F^2^ values of EL→ES, EL→MR, EL-→OrgT, MR→ES, and OrgT→ES are 0.210, 0.443, 0.451, 0.121, and 0.072, respectively, which proves the higher effect size has a strong association between variables as regression analysis. Figure 4 demonstrates the graphical representation of R^2^ and F^2^ values.

**TABLE 7:** R^2^, F^2^, and Q^2^ values.

Latent variables	R^2^	R^2Adj^	Q^2^	F^2^
ES	0.561	0.556	0.342	
MR	0.307	0.304	0.185	
OrgT	0.311	0.308	0.181	
EL→ES				0.210
EL→MR				0.443
EL-→OrgT				0.451
MR→ES				0.121
OrgT→ES				0.072

## Discussion

Significantly, over the years, various researchers have worked on ethics, but limited studies have comprehensively shed light on the role of EL in fostering employees’ job satisfaction (Vlachos et al., 2013). Hence, based on this research gap, this study illuminates the dynamic role of moral leadership in enhancing ES, MR, and OT in the healthcare industry of Macao. Ethical leaders play a fundamental role in boosting the individual’s and organization’s outcome. Indeed, in recent years, the growing significance of moral ethics has elevated the need for adopting EL for enhancing individuals’ output. Given the statement, the previous section (i.e., literature review) showed the potential relationship between the variables. However, this section aims to support the study results in the light of previous research. This section also discusses both the direct and indirect relationship in contrast to the fundamental findings of our study.

Ethical leaders’ moral character significantly influences individuals’ job satisfaction. Given the articulation, the study shows that EL enhances employees’ job satisfaction, thus guiding individual behavior (Negis Isik, 2020). This study indicates that H1 is supported. EL has a positive and significant impact on ES. The results are supported by advanced leadership that enhances organizational outcomes by reporting increased employees’ commitment and job satisfaction (Qing et al., 2020). Accordingly, the prior literature states that leadership constitutes a comprehensive communication model by making the Information Communication Technology (ICT) tools drive the business performance. Given the illustration, the research indicates that the novel leadership agenda (i.e., EL) has made the digital communication tools gain considerable importance, thereby influencing the behavioral elements of employees (Roman et al., 2019). Thus, H2 is supported. EL has a positive and significant impact on MR, and these results are supported by the more efficient the media channel is, the more ethical leaders influence team cohesion through moral cues (Sedrine et al., 2021).

Similarly, H3 is supported in this study. EL has a positive and significant impact on OT. Significantly, the findings of this study supported that moral leaders should look after the interest of both the internal and external stakeholders to maintain information transparency. Integrity, one of the crucial leadership traits, effectively motivates the leaders to share truthful information with firms’ stakeholders, subsequently enhancing organizational value and employee behavior (Ete et al., 2021). In addition to this, the study states that novel communication channels (e.g., social media platforms) improve employees’ satisfaction, thus building a solid employee-firm relationship (Erjavec et al., 2018). Thus, H4 is accepted and supported. MR has a positive and significant impact on ES. The results are confirmed and previous studies refer to videoconferencing as an innovative tool driving the firm’s communication (Lee et al., 2018) and employees’ satisfaction. Given the statement, the new influx of MR influences the organization’s communication with fostering ES (Chen and Chang, 2018). Moreover, H4a is supported and accepted, and MR significantly mediates the relationship between EL and ES supported by the prior literature (Özden et al., 2019).

Consistently, the research shows that perceived organizational communication suggests leaders build a transparent communication climate by fostering ES (Pološki Vokić et al., 2020). Thus, H5 is supported. OT has a positive and significant impact on ES. The study’s results are indicated that ES largely depends on the information available to them. Concerning the firms’ communication, exposure to false and inadequate data creates confusion for employees, thus making it difficult for them to manage the flooding of unnecessary and overburdened data (Yue et al., 2019). However, the H5a is supported and accepted, and OT mediates the relationship between EL and ES. The findings of this study revealed that OT is critical to developing an ethical climate in the organization. EL ensures the establishment of moral and transparent business practices, procedures, and communications, subsequently, achieving individual outcomes. The ethical environment enhances the employees’ perspective, thereby recording a positive response between EL and ES (Halbusi et al., 2021).

Accordingly, the study results suggest that leaders pay attention to the implementation of ethics, improving the organization’s affairs. Ethical behavior potentially influences employee action. Therefore, the study provides implications for the leaders to realize the importance of moral ethics in improving employees’ satisfaction. Furthermore, it also recommends increasing the amount of MR and OT for achieving favorable individual outcomes. This study provides a moral guide for today’s managers, leaders, scholars, and organizations for incorporating ethical practices, thus boosting employee work satisfaction.

## Conclusion

Since ancient times, EL has considerably gained experts’ attention. At present, the accelerated progress of moral ethics has made the leaders develop a strong moral character, fundamentally contributing to the organization’s welfare. However, raising the individual’s outcome has morally questioned the business practices, fostering the demand for EL. Indeed, the prior literature concludes EL to be a significant concept influencing the individual’s satisfaction. In the context of moral leadership, MR has also played a fundamental role in developing the organization’s communication process. The progressing MR drastically influences ES, thus demanding the leaders to emphasize developing an ethical climate of increased transparency.

Furthermore, the study also strengthens the role of MR and OT, thus ensuring favorable individual outcomes. MR and OT help leaders build an ethical climate, accelerating employees’ satisfaction and organizations’ progress. Perhaps, the study concluded that the impact of EL enhances employees’ satisfaction, thus promoting MR and OT to open the doors for the leaders to adopt novel communication tools for conveying the moral teachings.

Subsequently, to help advance our knowledge of EL, the study helps us understand the emerging role of moral leadership in today’s working environment. Results show that ethical leaders increase employees’ satisfaction at work. Furthermore, the study findings also provide empirical support to MR and OT. The mediating role of MR and OT illustrates a positive association between EL and ES. Overall, the research findings contribute to the growing knowledge of leadership virtues, thus stating its positive effect on ES, MR, and OT.

## Data Availability Statement

The raw data supporting the conclusions of this article will be made available by the authors, without undue reservation.

## Ethics Statement

Ethical review and approval were not required for the study on human participants in accordance with the local legislation and institutional requirements. The patients/participants provided their written informed consent to participate in this study.

## Author Contributions

This study was done by the author named in this article, and the author accept all liabilities resulting from claims which relate to this article and its contents.

## Conflict of Interest

The author declares that the research was conducted in the absence of any commercial or financial relationships that could be construed as a potential conflict of interest.

## Publisher’s Note

All claims expressed in this article are solely those of the authors and do not necessarily represent those of their affiliated organizations, or those of the publisher, the editors and the reviewers. Any product that may be evaluated in this article, or claim that may be made by its manufacturer, is not guaranteed or endorsed by the publisher.
